# Development and validation of the GLORIA-AF Stroke Weighted Risk Score for patients with atrial fibrillation

**DOI:** 10.1093/europace/euag236

**Published:** 2026-08-29

**Authors:** Yifan Xie, Wenhui Li, Yanda Meng, Tianjin Huang, Yalin Zheng, Menno V Huisman, Giuseppe Boriani, Tze-Fan Chao, Gregory Y H Lip

**Affiliations:** Institute of Life Course and Medical Sciences, University of Liverpool, William Henry Duncan Building, 6 West Derby Street, Liverpool L7 8TX, UK; Faculty of Environment, Science and Economy, University of Exeter, Exeter, UK; Institute of Life Course and Medical Sciences, University of Liverpool, William Henry Duncan Building, 6 West Derby Street, Liverpool L7 8TX, UK; Faculty of Environment, Science and Economy, University of Exeter, Exeter, UK; Bioengineering Program, Division of Biomedical Sciences, King Abdullah University of Science and Technology, Thuwal, Saudi Arabia; Faculty of Environment, Science and Economy, University of Exeter, Exeter, UK; Institute of Life Course and Medical Sciences, University of Liverpool, William Henry Duncan Building, 6 West Derby Street, Liverpool L7 8TX, UK; Department of Thrombosis and Hemostasis, Leiden University Medical Center, Leiden, The Netherlands; Cardiology Division, Department of Biomedical, Metabolic and Neural Sciences, University of Modena and Reggio Emilia, Modena, Italy; Cardiovascular Center, Taipei Veterans General Hospital, No. 201, Sec. 2, Shih-Pai Road, Taipei 112201, Taiwan; Institute of Life Course and Medical Sciences, University of Liverpool, William Henry Duncan Building, 6 West Derby Street, Liverpool L7 8TX, UK; Department of Clinical Medicine, Aalborg University, Aalborg, Denmark; Medical University of Bialystok, Bialystok, Poland

**Keywords:** Atrial fibrillation, Stroke prediction, Risk stratification, Oral anticoagulation, External validation

## Abstract

**Aims:**

Accurate stroke risk stratification is central to anticoagulation decision-making in patients with atrial fibrillation (AF), but conventional scores may not fully capture risk heterogeneity. We aimed to develop and externally validate an interpretable weighted score using a time-to-event framework.

**Methods and results:**

GLORIA-AF Phase II/III data were used to evaluate 17 baseline predictors using LASSO-penalized Cox regression with stability selection; coefficients were converted into integer weights. Performance was assessed using discrimination, calibration, integrated discrimination improvement (IDI), continuous net reclassification improvement (NRI), and decision curve analysis. External validation was performed in EORP-AF and APHRS-AF registries. Among 20 517 patients included in the derivation cohort [mean (SD) age, 69.9 (10.3) years; 9196 women (44.8%)], 487 (2.4%) had stroke, and 17 397 (84.8%) were receiving anticoagulation at baseline. Ten selected predictors formed a 0–23-point score. The derived score achieved a C-index of 0.661 (95% CI, 0.636–0.685), higher than CHA_2_DS_2_-VA (0.626; *P* < 0.001), CHA_2_DS_2_-VASc (0.615; *P* < 0.001), and the unweighted score (*P* = 0.016), with no significant difference from the full Cox or machine learning models. In external validation (8309 patients; 147 strokes), the C-index was 0.652 (95% CI, 0.614–0.690) vs. 0.616 for CHA_2_DS_2_-VA (95% CI, 0.598–0.634; *P* < 0.001). IDI/NRI, calibration, and decision curve analyses supported improved risk differentiation, close calibration, and generally greater net benefit than CHA_2_DS_2_-VA. The score retained higher discrimination than CHA_2_DS_2_-VA among patients without baseline anticoagulation (*P* < 0.001).

**Conclusion:**

The GLORIA-AF Stroke Weighted Risk Score provides risk refinement beyond CHA_2_DS_2_-VA while retaining discrimination consistent with more complex models. External validation supports its transportability and potential adjunctive role in guideline-directed thromboembolic risk assessment.

## Graphical Abstract

**Graphical Abstract:**
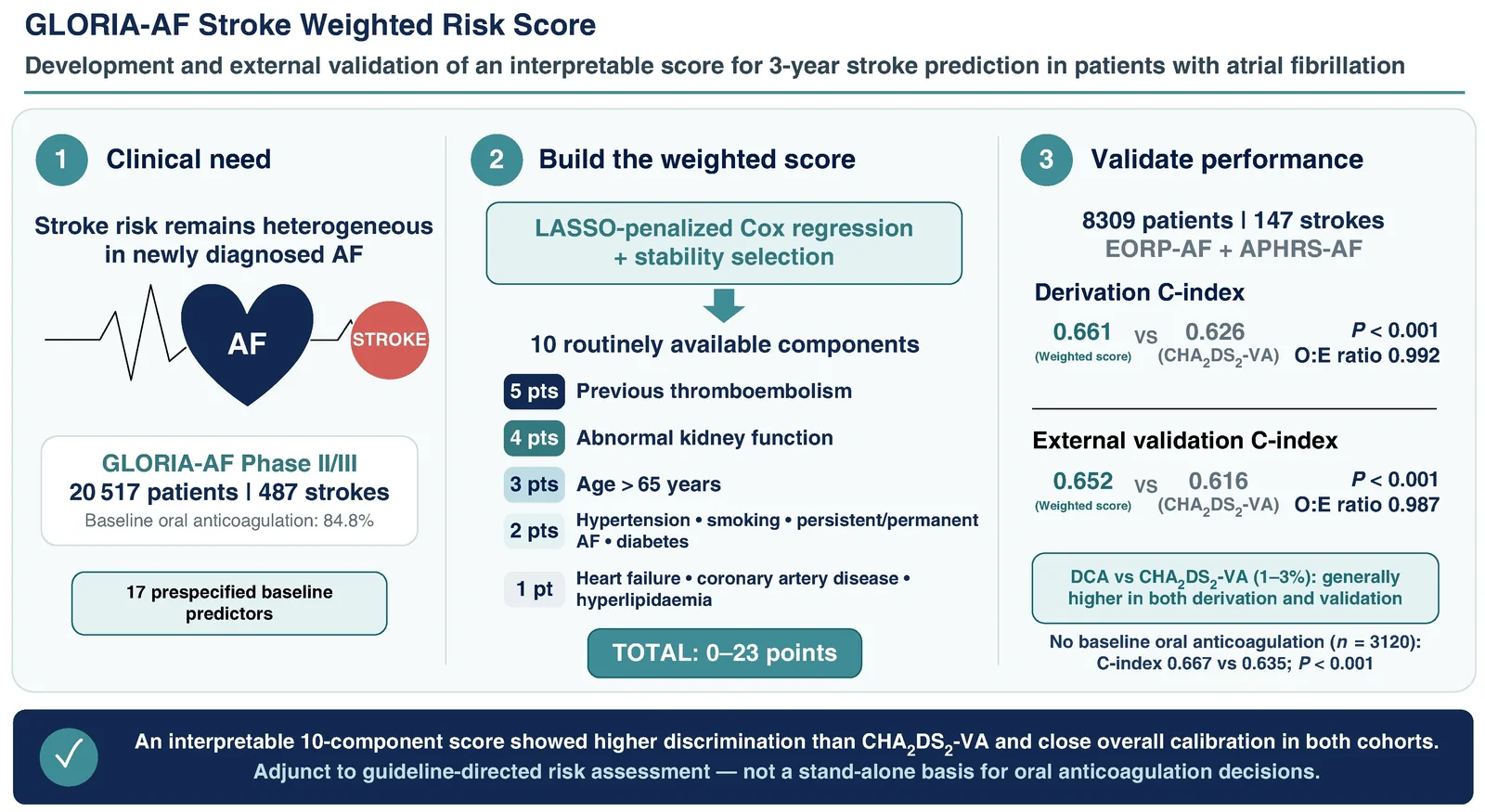

What’s new?

- The GLORIA-AF Stroke Weighted Risk Score translates 10 routinely available baseline predictors into an interpretable 0–23-point tool, combining LASSO-penalized Cox regression, stability selection, and coefficient-based integer weighting within a time-to-event framework.
- The weighted score showed higher discrimination than CHA_2_DS_2_-VA, CHA_2_DS_2_-VASc, and the corresponding unweighted score. Its discrimination did not differ significantly from that of the evaluated more complex regression and machine learning models.
- Independent validation in European and Asia-Pacific registries demonstrated close calibration, incremental risk separation, and generally favourable decision-analytic performance relative to CHA_2_DS_2_-VA, supporting transportability across geographically distinct AF populations.
- The score retained higher discrimination than CHA_2_DS_2_-VA among patients not receiving oral anticoagulation at baseline, supporting its potential role as an adjunctive tool for refined risk assessment when anticoagulation is being considered.

## Introduction

Atrial fibrillation (AF) is the most common sustained cardiac arrhythmia and is associated with an increased risk of ischaemic stroke.^1–3^ When stroke occurs in patients with AF, it is often more severe and associated with greater disability and mortality than stroke in patients without AF.^4,5^ However, thromboembolic risk is heterogeneous and varies substantially according to individual clinical characteristics, comorbidities, and AF burden.^6,7^ Oral anticoagulation (OAC) treatment is the cornerstone of stroke prevention in patients with AF, but treatment decisions require an individualized assessment of thromboembolic risk in the context of bleeding risk and patient preference.^4,8^ Accurate and clinically usable stroke risk stratification therefore remains central to anticoagulation decision-making and longitudinal AF care.

The CHA_2_DS_2_-VASc score has historically been the most widely used guideline-endorsed tool for stroke risk stratification in patients with AF.^7^ More recently, European guidance has adopted the non-sex CHA_2_DS_2_-VA score for thromboembolic risk assessment, reflecting the view that female sex is better considered as a risk modifier than as an independent treatment-triggering risk factor.^9^ Nevertheless, both CHA_2_DS_2_-VASc and CHA_2_DS_2_-VA retain a simple factor-counting structure, and their discriminatory performance remains moderate, with C-statistics in external validation studies generally ranging from ∼0.60 to 0.65.^10,11^ This limited performance may reflect omission of clinically relevant variables and the assumption that included risk factors contribute equally to risk.^12,13^ More complex statistical and machine learning models may improve predictive performance, but these gains are often offset by reduced transparency, greater implementation burden, and limited usability at the point of care.^14–16^

The Global Registry on Long-Term Oral Antithrombotic Treatment in Patients With Atrial Fibrillation (GLORIA-AF) is a large, prospective, multinational registry of consecutively enrolled patients with newly diagnosed AF (ClinicalTrials.gov ID: NCT01468701).^17,18^ The registry’s systematic collection of demographic characteristics, lifestyle factors, comorbidities, and treatment information provides a suitable foundation for developing a clinically usable weighted risk score.

The objectives of this study were (i) to derive the GLORIA-AF Stroke Weighted Risk Score using routinely available baseline variables; (ii) to compare its discrimination, calibration, and decision-analytic net benefit with CHA_2_DS_2_-VA, CHA_2_DS_2_-VASc, an unweighted GLORIA-AF score, the full multivariable Cox regression, and more complex machine learning models; and (iii) to externally validate the derived score in independent European and Asia-Pacific AF registries and assess its robustness in patients receiving no baseline OAC treatment.

## Methods

### Study design and population

This prognostic study was a secondary analysis using Phase II/III data from the GLORIA-AF, a prospective, multinational registry programme originally designed to assess real-world antithrombotic treatment patterns and outcomes in patients with newly diagnosed non-valvular AF.^17,18^ Consecutive patients aged ≥18 years and with a CHA_2_DS_2_-VASc score of ≥1 were enrolled in routine clinical practice. In this study, patients were eligible if they had complete baseline data for prespecified predictors and available follow-up for stroke event. Patients with missing or invalid information in one or more required variables were excluded. Follow-up was measured from baseline to the earliest occurrence of stroke, death, loss to follow-up or withdrawal, or 36 months.

Ethics approvals were received from local institutional review boards, and all patients provided their handwritten informed consents. The study was conducted in accordance with the Good Clinical Practice and the Declaration of Helsinki. This study followed the Transparent Reporting of a Multivariable Prediction Model for Individual Prognosis or Diagnosis (TRIPOD) reporting guidelines.

### Candidate predictors and outcome

A total of 17 candidate predictors were prespecified based on clinical relevance, data availability, and feasibility for use in a clinical risk score: age, sex, heart failure, hypertension, previous thromboembolism, vascular disease, diabetes, body mass index (BMI), ethnicity, smoking history, alcohol consumption, type of AF, hyperlipidaemia, coronary artery disease, previous bleeding, respiratory disease, and abnormal kidney function. To support model development, all predictors were defined at baseline and transformed into binary indicators based on guidelines and prior literature; detailed definitions are provided in Supplementary material online, *Table S1*.

The primary outcome was stroke, defined as an acute focal neurologic deficit of presumed vascular origin lasting at least 24 h or resulting in death.^19^ The CHA_2_DS_2_-VASc and CHA_2_DS_2_-VA scores were calculated according to their original methodology; no modified version of the score was used in this study.^7^

### Score derivation and statistical analysis

Baseline characteristics were summarized using original clinical variables as mean (SD) for continuous variables and no. (%) for categorical variables. Comparisons by stroke status used *t*-test or *χ*^2^ test for descriptive purpose only and were not used for predictor selection. Score derivation used the binary-transformed candidate predictors.

The score was derived in two stages. First, LASSO-penalized Cox proportional hazards regression with stability selection was applied to the prespecified binary candidate predictors.^20,21^ The proportional hazards assumption for the Cox model was assessed using Schoenfeld residuals.^22^ In this approach, LASSO penalization shrinks less informative coefficients towards zero, and predictors with a stability selection probability of ≥0.80 were retained for score derivation; the mathematical formulation of penalized regression with stability selection is provided in Supplementary material online, *Methods*. Second, retained predictors were entered into a multivariable Cox proportional regression, and coefficients were rescaled according to their relative magnitude and converted into point values from 1 to 5 (see Supplementary material online, *Methods*), allowing the final score to be calculated by summing points for risk factors present in an individual patient.^23^

Model performance was evaluated in terms of discrimination, calibration, and decision-analytic net benefit. Discrimination was quantified using the Harrell’s concordance index (C-index) with 95% confidence interval (CI) and compared with the CHA_2_DS_2_-VA score, the CHA_2_DS_2_-VASc score, an unweighted GLORIA-AF stroke score based on the same selected predictor set, the full multivariable Cox regression, and machine learning models [random forest (RF) and extreme gradient boosting (XGBoost)] trained on the same predictor set with hyperparameters optimized using cross-validation.^7,24–27^ Pairwise differences in C-index were evaluated using Wald tests. Incremental discrimination over the CHA_2_DS_2_-VA score was further assessed using integrated discrimination improvement (IDI) and continuous net reclassification improvement (NRI) with 95% CIs; event and non-event NRI components were also reported.^28,29^ Discrimination slopes were calculated for both scores and formally compared. Overall calibration was summarized by the observed-to-expected (O:E) ratio.^30^ Calibration plots compared predicted and observed stroke risk.^31^ Decision curve analysis compared the derived score, CHA_2_DS_2_-VA, treat-all, and treat-none strategies over threshold probabilities of 1.0–3.0%.^32^ Net benefit reflects the clinical utility of a model-guided strategy by balancing true- and false-positive classifications. All analyses were performed using R, version 4.3.1 (R Foundation for Statistical Computing).

### External validation and sensitivity analysis

External validation was performed in the combined EURObservational Research Programme Atrial Fibrillation (EORP-AF) Registry and Asia-Pacific Heart Rhythm Society Atrial Fibrillation (APHRS-AF) Registry.^33,34^ The GLORIA-AF Stroke Weighted Risk Score was applied to the external validation cohort without refitting or recalibration to assess its transportability across geographically distinct AF populations. Discrimination, calibration, and decision-analytic net benefit were evaluated and compared using the same procedures as in the derivation cohort.

To assess the prognostic performance of the GLORIA-AF Stroke Weighted Risk Score among patients not receiving OAC at baseline, a sensitivity analysis was performed in this subgroup, defined as no NOAC or VKA use at baseline. The derived score was applied without refitting or recalibration, and discrimination was assessed using C-index with 95% CIs, following the same procedures as in the primary analysis. Pairwise differences in C-index were evaluated using Wald tests.

## Results

### Study population and score derivation

The GLORIA-AF Phase II/III source dataset comprised 36 617 patients. After application of the complete-case requirements, 16 100 (44.0%) patients were excluded because of missing or invalid information in at least one required predictor or stroke follow-up. Variable-level missingness was reported in Supplementary material online, *Table S2*. Among 20 517 patients (56.0%) included in this analysis [mean (SD) age, 69.9 (10.3) years; 9196 women (44.8%)], 487 (2.4%) had stroke during 3-year follow-up. Baseline characteristics are summarized in *Table 1*.

**Table 1:** Baseline characteristics by stroke outcome

Variable	Overall (*n* = 20 517)	Stroke (*n* = 487)	No stroke (*n* = 20 030)	*P* value^a^
Age, mean (SD), y	69.9 (10.3)	73.3 (9.0)	69.8 (10.3)	<0.001
Female, no. (%)	9196 (44.8)	211 (43.3)	8985 (44.9)	0.532
Heart failure, no. (%)	4513 (22.0)	122 (25.1)	4391 (21.9)	0.111
Hypertension, no. (%)	15 620 (76.1)	398 (81.7)	15 222 (76.0)	0.004
Previous thromboembolism, no. (%)	2971 (14.5)	141 (29.0)	2830 (14.1)	<0.001
Vascular disease, no. (%)	2558 (12.5)	68 (14.0)	2490 (12.4)	0.346
Diabetes, no. (%)	4842 (23.6)	140 (28.7)	4702 (23.5)	0.008
BMI, mean (SD)	28.9 (6.3)	27.9 (5.6)	28.9 (6.3)	<0.001
Ethnicity, no. (%)				<0.001
White	15 294 (74.5)	323 (66.3)	14 971 (74.7)	
Arab or Middle Eastern	273 (1.3)	3 (0.6)	270 (1.3)	
Asian	3860 (18.8)	138 (28.3)	3722 (18.6)	
Black or Afro-Caribbean	373 (1.8)	12 (2.5)	361 (1.8)	
Other	717 (3.5)	11 (2.3)	706 (3.5)	
Smoking history, no. (%)				0.004
Never	12 182 (59.4)	259 (53.2)	11 923 (59.5)	
Former	6380 (31.1)	164 (33.7)	6216 (31.0)	
Current	1955 (9.5)	64 (13.1)	1891 (9.4)	
Alcohol intake, no. (%)				0.438
None	9508 (46.3)	243 (49.9)	9265 (46.3)	
<1 drink/wk	5262 (25.6)	116 (23.8)	5146 (25.7)	
1–7 drinks/wk	4314 (21.0)	94 (19.3)	4220 (21.1)	
≥8 drinks/wk	1433 (7.0)	34 (7.0)	1399 (7.0)	
Type of AF, no. (%)				0.054
Paroxysmal	11 636 (56.7)	251 (51.5)	11 385 (56.8)	
Persistent	6895 (33.6)	187 (38.4)	6708 (33.5)	
Permanent	1986 (9.7)	49 (10.1)	1937 (9.7)	
Hyperlipidaemia, no. (%)	8517 (41.5)	233 (47.8)	8284 (41.4)	0.005
Coronary artery disease, no. (%)	4042 (19.7)	118 (24.2)	3924 (19.6)	0.013
Previous bleeding, no. (%)	1126 (5.5)	32 (6.6)	1094 (5.5)	0.337
Respiratory disease, no. (%)	2302 (11.2)	63 (12.9)	2239 (11.2)	0.253
Abnormal kidney function, no. (%)	331 (1.6)	16 (3.3)	315 (1.6)	0.005
Baseline OAC treatment, no. (%)				<0.001
None	3120 (15.2)	109 (22.4)	3011 (15.0)	
NOAC	13 569 (66.1)	275 (56.5)	13 294 (66.4)	
VKA	3828 (18.7)	103 (21.1)	3725 (18.6)	
CHA_2_DS_2_-VA score, mean (SD)	2.7 (1.4)	3.4 (1.5)	2.7 (1.4)	<0.001
CHA_2_DS_2_-VASc score, mean (SD)	3.2 (1.5)	3.8 (1.6)	3.1 (1.5)	<0.001

^a^ *P* values were calculated for descriptive comparisons and were not used for predictor selection.

SD, standard deviation; BMI, body mass index; AF, atrial fibrillation; OAC, oral anticoagulant; NOAC, non-vitamin K antagonist oral anticoagulant; VKA, vitamin K antagonist.

Of the 17 prespecified candidate predictors, 10 met the stability selection criterion and were retained in the final multivariable Cox model. There was no evidence of violation of the proportional hazards assumption (*P* = 0.688). The highest weight was assigned to previous thromboembolism (5 points), followed by abnormal kidney function (4 points); the complete scoring algorithm was presented in *Table 2*. The total score ranged from 0 to 23 points; the full score calculation is provided in Supplementary material online, *Methods*. Mean score was higher among patients with stroke than among those without stroke [9.7 (3.2) vs. 7.4 (3.1); *P* < 0.001].

**Table 2:** Full scoring algorithm of the GLORIA-AF Stroke Weighted Risk Score

Variable^a^	Selection probability by LASSO, %	β coefficient	Assigned points
Previous thromboembolism	100	0.858	5
Abnormal kidney function	100	0.632	4
Age > 65 years	100	0.524	3
Hypertension	99	0.276	2
Smoking history	100	0.247	2
Persistent or permanent AF	94	0.210	2
Diabetes	92	0.194	2
Heart failure	85	0.183	1
CAD	87	0.090	1
Hyperlipidaemia	82	0.088	1

^a^Definitions of binary-transformed risk factors are provided in Supplementary material online, *Table S1*.

AF, atrial fibrillation; LASSO, least absolute shrinkage and selection operator; CAD, coronary artery disease.

### Model performance in the derivation cohort

The GLORIA-AF Stroke Weighted Risk Score achieved a C-index of 0.661 (95% CI, 0.636–0.685) for 3-year stroke prediction in the derivation cohort, which was significantly higher than CHA_2_DS_2_-VA (C-index, 0.626; 95% CI, 0.613–0.639; *P* < 0.001) and CHA_2_DS_2_-VASc (C-index, 0.615; 95% CI, 0.600–0.630; *P* < 0.001). The weighted score also outperformed the unweighted GLORIA-AF stroke score (C-index, 0.643; 95% CI, 0.619–0.666; *P* = 0.016). In contrast, its discrimination did not differ significantly from that of the full multivariable Cox model (C-index, 0.669; 95% CI, 0.645–0.693; *P* = 0.532), RF (C-index, 0.659; 95% CI, 0.629–0.688; *P* = 0.407), or XGBoost (C-index, 0.667; 95% CI, 0.632–0.702; *P* = 0.412).

Compared with CHA_2_DS_2_-VA, the GLORIA-AF Stroke Weighted Risk Score showed an IDI of 0.0052 (95% CI, 0.0034–0.0071) and a total continuous NRI of 0.1830 (95% CI, 0.0848–0.2745), comprising an event NRI of 0.1367 (95% CI, 0.0443–0.2239) and a non-event NRI of 0.0463 (95% CI, 0.0262–0.0663). The discrimination slope was 0.0123 for the GLORIA-AF Stroke Weighted Risk Score and 0.0071 for CHA_2_DS_2_-VA, corresponding to a 73.2% relative increase (*P* < 0.001) (see Supplementary material online, *Table S3*).

The O:E ratio was 0.992, indicating good overall agreement between observed and predicted 3-year stroke risk. Graphical calibration plots similarly showed close agreement across the observed risk range (*Figure 1*). Decision curve analysis showed consistently greater net benefit for the weighted score than for CHA_2_DS_2_-VA and the treat-all and treat-none strategies across the prespecified threshold probability range of 1.0–3.0% (*Figure 2*).

**Figure 1:**
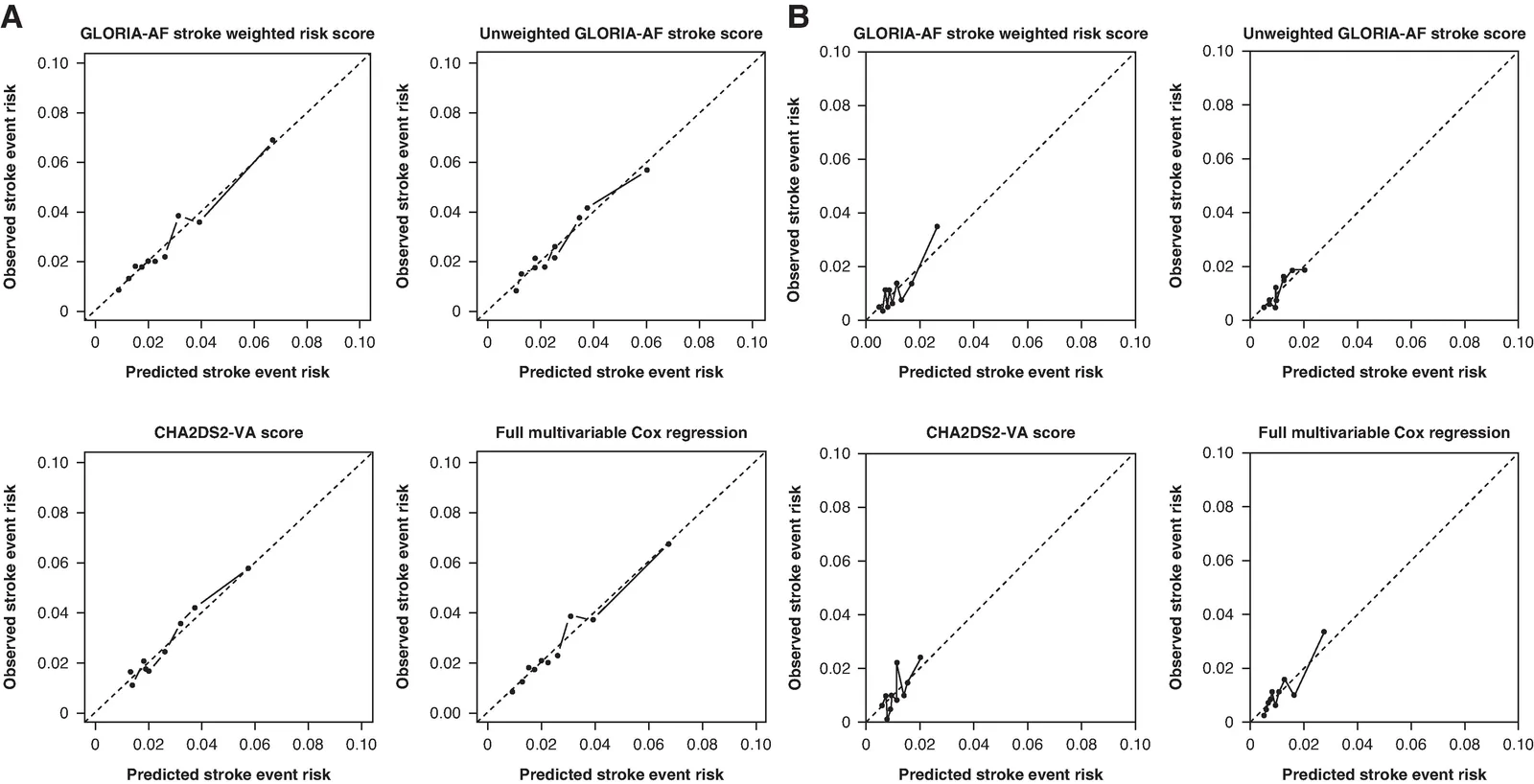
Calibration plots for the GLORIA-AF Stroke Weighted Risk Score and comparator models. Calibration plots for the GLORIA-AF Stroke Weighted Risk Score, the CHA_2_DS_2_-VA score, an unweighted GLORIA-AF stroke score, and the full multivariable Cox regression. Panel (*A*) presents the derivation cohort, and panel (*B*) presents the external validation cohort. The *x*-axis represents the predicted stroke risk, and the *y*-axis represents the corresponding observed risk. Black points denote observed risk estimates plotted against the corresponding predicted risks. The dashed 45-degree line represents perfect agreement between predicted and observed risks.

**Figure 2:**
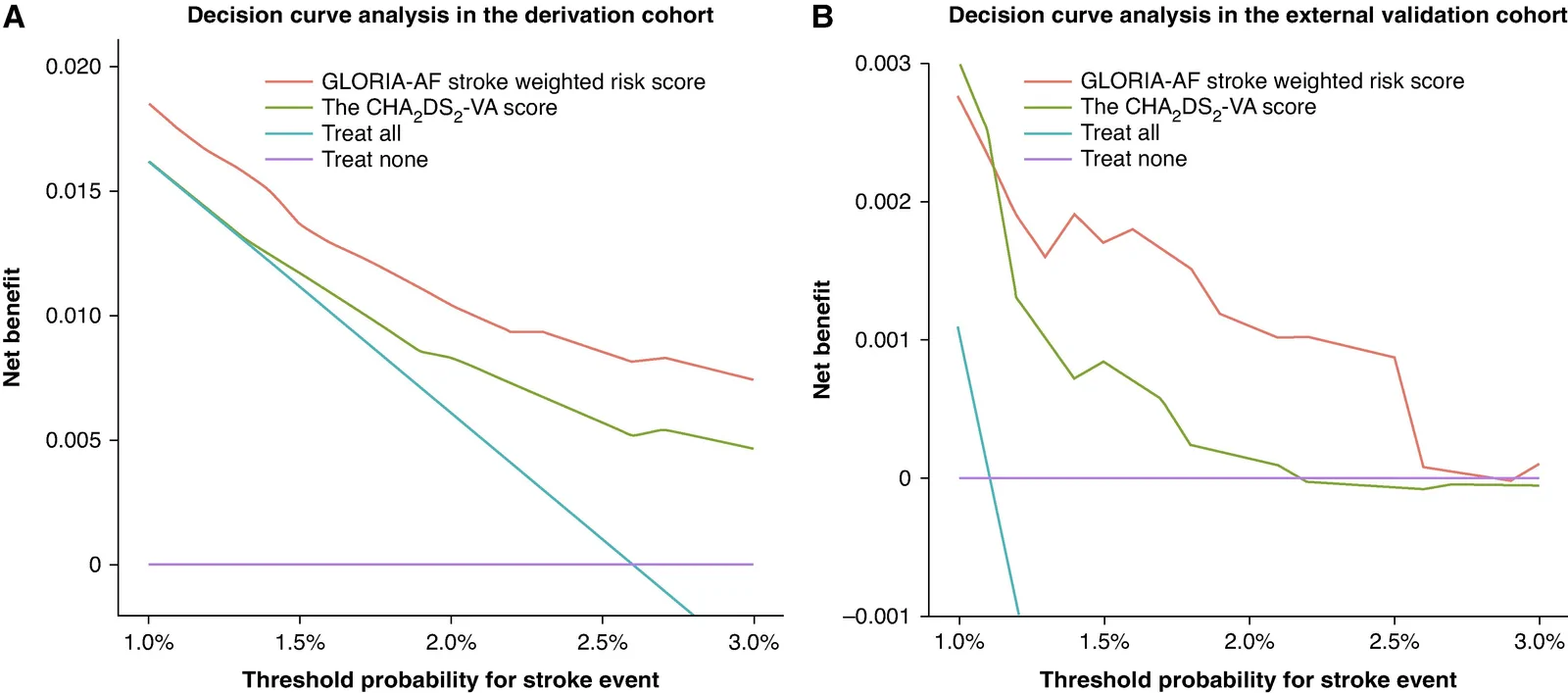
Decision curve analysis of the GLORIA-AF Stroke Weighted Risk Score and comparator models. Decision curve analysis evaluates the clinical net benefit of the GLORIA-AF Stroke Weighted Risk Score and comparator models for stroke prediction. Panel (*A*) presents the derivation cohort, and panel (*B*) presents the external validation cohort. Net benefit is shown across threshold probabilities ranging from 1.0 to 3.0%. At a given threshold probability, a higher net benefit indicates greater potential clinical utility.

### External validation and sensitivity analysis

The source external validation cohort comprised 15 762 patients, including 11 096 from EORP-AF and 4666 from APHRS-AF. After application of the complete-case requirements, 7453 (47.3%) patients were excluded because of missingness. Among the 8309 (52.7%) patients included [age 68.8 (11.4) years; 3135 women (37.7%)], 147 (1.8%) had stroke. Baseline characteristics are summarized in Supplementary material online, *Table S4*.

In the external validation cohort, the GLORIA-AF Stroke Weighted Risk Score achieved a C-index of 0.652 (95% CI, 0.614–0.690), significantly higher than CHA_2_DS_2_-VA (C-index, 0.616; 95% CI, 0.598–0.634; *P* < 0.001) and CHA_2_DS_2_-VASc (C-index, 0.612; 95% CI, 0.587–0.637; *P* < 0.001) (*Table 3*). Compared with CHA_2_DS_2_-VA, the weighted score showed an IDI of 0.0034 (95% CI, 0.0002–0.0067) and a higher discrimination slope (0.0061 vs. 0.0027; *P* = 0.043); detailed IDI/NRI results are provided in Supplementary material online, *Table S3*. The O:E ratio was 0.987, indicating close overall agreement between observed and predicted risks, which was also supported by the calibration plot (*Figure 1*). Decision curve analysis showed generally greater net benefit for the weighted score than for CHA_2_DS_2_-VA and the treat-all and treat-none strategies across most of the prespecified threshold probability range of 1.0–3.0% (*Figure 2*).

**Table 3:** Model discrimination in derivation cohort, external validation cohort, and sensitivity analysis

Cohort and model	C-index (95% CI)	*P* value^a^
Derivation cohort		
GLORIA-AF Stroke Weighted Risk Score	0.661 (0.636–0.685)	Reference
CHA_2_DS_2_-VASc	0.615 (0.600–0.630)	<0.001
CHA_2_DS_2_-VA	0.626 (0.613–0.639)	<0.001
Unweighted GLORIA-AF stroke score	0.643 (0.619–0.666)	0.016
Full multivariable Cox regression	0.669 (0.645–0.693)	0.532
Random forest	0.659 (0.629–0.688)	0.407
XGBoost	0.667 (0.632–0.702)	0.412
External validation cohort		
GLORIA-AF Stroke Weighted Risk Score	0.652 (0.614–0.690)	Reference
CHA_2_DS_2_-VASc score	0.612 (0.587–0.637)	<0.001
CHA_2_DS_2_-VA score	0.616 (0.598–0.634)	<0.001
Unweighted GLORIA-AF stroke score	0.637 (0.612–0.662)	<0.001
Full multivariable Cox regression	0.659 (0.623–0.695)	0.548
Sensitivity analysis		
GLORIA-AF Stroke Weighted Risk Score	0.667 (0.636–0.697)	Reference
CHA_2_DS_2_-VA score	0.635 (0.621–0.649)	<0.001

^a^ *P* values of pairwise differences in C-index between the GLORIA-AF Stroke Weighted Risk Score and comparator models were evaluated using Wald tests.

C-index, concordance index; XGBoost, extreme gradient boosting.

Among 3120 (15.2%) patients who were not receiving OAC treatment at baseline in the derivation cohort, 109 (3.5%) had stroke during follow-up. In this subgroup, the GLORIA-AF Stroke Weighted Risk Score achieved a C-index of 0.667 (95% CI, 0.636–0.697), significantly higher than that of CHA_2_DS_2_-VA (C-index, 0.635; 95% CI, 0.621–0.649; *P* < 0.001) (*Table 3*).

## Discussion

In this prognostic study using data from a large, prospective, multinational registry of patients with newly diagnosed AF, we developed the GLORIA-AF Stroke Weighted Risk Score using a time-to-event framework and evaluated its performance in independent European and Asia-Pacific cohorts. The resulting weighted score comprised 10 components, ranged from 0 to 23 points, and demonstrated higher discrimination than CHA_2_DS_2_-VA, CHA_2_DS_2_-VASc, and the corresponding unweighted GLORIA-AF score in both the derivation and external validation cohorts. Importantly, its discrimination did not differ significantly from that of the full multivariable Cox model in either cohort, suggesting that conversion of the regression coefficients into integer weights preserved the discriminative performance of the full model. Its discrimination also did not differ significantly from that of the RF and XGBoost models in the derivation cohort. The weighted score showed close overall agreement between observed and predicted risks in both the derivation and external validation cohorts and generally provided greater decision-analytic net benefit than CHA_2_DS_2_-VA across most of the prespecified threshold probability range. Collectively, these findings suggest that appropriately weighting routinely available clinical predictors can provide incremental refinement of stroke risk stratification while preserving interpretability and practical usability.

The principal contribution of the GLORIA-AF Stroke Weighted Risk Score lies in its ability to balance practical simplicity with predictive refinement. Conventional clinical scores are straightforward to calculate but generally use a limited set of fixed weights that may not fully capture differences in prognostic strength among individual risk factors.^7,9,35^ This weighted score was developed from clinically prespecified candidate predictors using LASSO-penalized Cox regression with stability selection, and coefficient-based integer weighting, thereby translating the relative prognostic contributions of the selected predictors into a familiar point-based format.^20,23^ The underlying time-to-event framework incorporated the timing of stroke while accounting for censoring, allowing all participants, including those with less than 3 years of observation, to contribute information over their available follow-up. This approach is particularly well suited to multinational registry data with varying durations of observation. Despite being based on the same selected predictors, the weighted score achieved a significantly higher C-index than the corresponding unweighted score in both cohorts, supporting the added discriminatory value of differential weighting beyond predictor selection alone. The absence of statistically significant differences in discrimination between the weighted score and either the full multivariable Cox regression model or the machine learning models suggests that converting the regression coefficients into integer points retained much of the discriminative information contained in the full Cox model, yielding an interpretable point-based score with discrimination broadly consistent with that of the more complex models.

Model performance should not be judged on the C-index alone. We therefore further evaluated incremental risk separation and reclassification using IDI, discrimination slope, and continuous NRI, alongside calibration and decision curve analyses.^28–32^ These analyses indicated greater risk separation in both cohorts, although the reclassification evidence was more conclusive in the derivation cohort than in the external validation cohort. Calibration analyses supported close agreement between observed and predicted risks at the evaluated prediction horizons. Decision curve analysis, which evaluates potential clinical utility by balancing true-positive and false-positive classifications at selected risk thresholds, suggested greater net benefit for the weighted score across the evaluated threshold range. Nevertheless, net benefit reflects the value of a model-guided decision strategy rather than direct benefit to an individual patient, and the evaluated thresholds should not be regarded as validated OAC treatment cut-offs. Taken together, these complementary findings provide evidence that the weighted score offers incremental prognostic value beyond CHA_2_DS_2_-VA across multiple dimensions of model performance, supporting its potential utility for more refined stroke risk stratification.

The GLORIA-AF Stroke Weighted Risk Score should be interpreted in the context of prior stroke prediction models for patients with AF. Models such as ATRIA, GARFIELD-AF, ABC-stroke, and many regional risk tools have demonstrated that stroke prediction in patients with AF can be refined beyond conventional factor-counting schemes, but often at the cost of broader range of clinical variables, biomarkers, or more complex calculation methods.^10,11,36–39^ The derived score offers a clinically practical alternative by incorporating broader prognostic information than CHA_2_DS_2_-VA and CHA_2_DS_2_-VASc while retaining a straightforward point-based calculation based entirely on routinely available clinical variables.

External validation is particularly important for prognostic models because performance may decline outside the derivation setting. The external validation cohort combined independent European and Asia-Pacific AF registries, allowing assessment across populations with different clinical characteristics, treatment patterns, and regional risk profiles.^33,34^ In this independent setting, the weighted score maintained higher discrimination than conventional scores, while also demonstrating good calibration and favourable decision-analytic performance across most of the evaluated threshold range. The convergence of these findings across complementary performance domains and geographically distinct populations supports the transportability of the score beyond the derivation cohort. Because the main reason for estimating stroke risk in patients with AF is to inform decisions regarding OAC initiation, it is important to establish whether the derived score remains informative among patients not receiving anticoagulation at the time of assessment.^7^ Given that most patients in the derivation cohort were already receiving OAC at baseline, the sensitivity analysis specifically evaluated the performance of the weighted score in this clinically relevant subgroup. The score retained higher discrimination than CHA_2_DS_2_-VA, supporting its robustness and potential value as an adjunctive risk refinement tool when OAC initiation is being considered.

The GLORIA-AF Stroke Weighted Risk Score should be regarded primarily as an adjunctive prognostic tool for more refined stratification of future stroke risk in the context of routine clinical care. It is intended to complement CHA_2_DS_2_-VA within guideline-directed thromboembolic risk assessment by providing more granular prognostic information and potentially supporting risk communication, rather than to replace guideline-based assessment or serve as a stand-alone basis for initiating, continuing, or discontinuing OAC. The score does not estimate treatment effects or counterfactual stroke risk under the sustained absence of anticoagulation. Accordingly, the selected predictors should be interpreted as prognostic markers rather than causal determinants; neither their inclusion nor their assigned weights should be taken to imply biological causality or direct therapeutic relevance. Prospective impact studies are needed to establish whether use of the score improves decision-making or patient outcomes before its adoption in routine clinical practice. As the score is agnostic to OAC use/type, it could potentially be applied to address the residual risk of events even amongst anticoagulated patients.

### Limitations

Several limitations should be acknowledged. First, converting non-binary baseline variables into binary indicators during score development improved usability but may have reduced predictor granularity and resulted in some loss of prognostic information. Second, stroke outcomes in the EORP-AF and APHRS-AF cohorts were prospectively recorded by investigators at participating sites rather than centrally adjudicated, which may have introduced some uncertainty in outcome ascertainment. Third, registry participants may differ in case mix from unselected all-comer populations with AF, which should be considered when generalizing the findings to other settings. Fourth, the external validation cohorts had shorter available follow-up than GLORIA-AF, providing less extensive evidence regarding longer-term absolute-risk performance. Finally, because the complete-case approach required complete information on the outcome, follow-up, and all candidate predictors, a proportion of patients were excluded from the source cohorts, which may have affected the representativeness of the resulting analytic cohorts.

## Conclusions

The GLORIA-AF Stroke Weighted Risk Score is a clinically interpretable tool for predicting stroke risk in patients with AF, achieving higher discrimination than CHA_2_DS_2_-VA while showing no statistically significant difference from more complex regression and machine learning models. External validation in independent European and Asia-Pacific AF registries supported its transportability across geographically distinct populations. The score may therefore serve as an adjunctive prognostic tool for more granular stroke risk stratification within guideline-directed thromboembolic risk assessment at or near AF diagnosis.

## Supplementary Material

euag236_Supplementary_Data

## Data Availability

Data supporting this study was from the data contributors Boehringer Ingelheim, and were made and are available through Vivli, Inc. Access was provided after a proposal was approved by an independent review committee identified for this purpose and after receipt of a signed data sharing agreement.

## References

[1] Cheng S, He J, Han Y, Han S, Li P, Liao H et al Global burden of atrial fibrillation/atrial flutter and its attributable risk factors from 1990 to 2021. Europace 2024;26:euae195. 10.1093/europace/euae195 PMC11287210 38984719

[2] Borre ED, Goode A, Raitz G, Shah B, Lowenstern A, Chatterjee R et al Predicting thromboembolic and bleeding event risk in patients with non-valvular atrial fibrillation: a systematic review. Thromb Haemost 2018;118:2171–87. 10.1055/s-0038-1675400 PMC6754740 30376678

[3] Tan SCW, Tang ML, Chu H, Zhao YT, Weng C. Trends in global burden and socioeconomic profiles of atrial fibrillation and atrial flutter: insights from the global burden of disease study 2021. CJC Open 2025;7:247–58. 10.1016/j.cjco.2024.11.017 PMC11963203 40182400

[4] Chao TF, Potpara TS, Lip GYH. Atrial fibrillation: stroke prevention. Lancet Reg Health Eur 2024;37:100797. 10.1016/j.lanepe.2023.100797 PMC10867001 38362551

[5] Marini C, De Santis F, Sacco S, Russo T, Olivieri L, Totaro R et al Contribution of atrial fibrillation to incidence and outcome of ischemic stroke: results from a population-based study. Stroke 2005;36:1115–9. 10.1161/01.STR.0000166053.83476.4a 15879330

[6] Go AS, Reynolds K, Yang J, Gupta N, Lenane J, Sung SH et al Association of burden of atrial fibrillation with risk of ischemic stroke in adults with paroxysmal atrial fibrillation: the KP-RHYTHM study. JAMA Cardiol 2018;3:601–8. 10.1001/jamacardio.2018.1176 PMC6145663 29799942

[7] Lip GY, Nieuwlaat R, Pisters R, Lane DA, Crijns HJ. Refining clinical risk stratification for predicting stroke and thromboembolism in atrial fibrillation using a novel risk factor-based approach: the euro heart survey on atrial fibrillation. Chest 2010;137:263–72. 10.1378/chest.09-1584 19762550

[8] Gorog DA, Gue YX, Chao TF, Fauchier L, Ferreiro JL, Huber K et al Assessment and mitigation of bleeding risk in atrial fibrillation and venous thromboembolism: executive summary of a European and Asia-Pacific expert consensus paper. Thromb Haemost 2022;122:1625–52. 10.1055/s-0042-1750385 35793691

[9] Van Gelder IC, Rienstra M, Bunting KV, Casado-Arroyo R, Caso V, Crijns HJGM et al 2024 ESC guidelines for the management of atrial fibrillation developed in collaboration with the European association for cardio-thoracic surgery (EACTS). Eur Heart J 2024;45:3314–414. 10.1093/eurheartj/ehae176 39210723

[10] Krittayaphong R, Winijkul A, Sairat P, Lip GYH. Predicting the absolute risk of ischemic stroke in Asian patients with atrial fibrillation: comparing the COOL-AF risk score with CARS/mCARS models for absolute risk and the CHA2DS2-VASc score. J Clin Med 2023;12:2449. 10.3390/jcm12072449 PMC10095200 37048533

[11] Krittayaphong R, Kanjanarutjawiwat W, Wisaratapong T, Lip GYH. Improving risk prediction for death, stroke and bleeding in Asian patients with atrial fibrillation. Eur J Clin Invest 2023;53:e13886. 10.1111/eci.13886 36197442

[12] Krittayaphong R, Winijkul A, Sairat P. Left atrial diameter in the prediction of thromboembolic event and death in atrial fibrillation. J Clin Med 2022;11:1838. 10.3390/jcm11071838 PMC8999165 35407446

[13] Friberg L, Rosenqvist M, Lip GY. Evaluation of risk stratification schemes for ischaemic stroke and bleeding in 182 678 patients with atrial fibrillation: the Swedish atrial fibrillation cohort study. Eur Heart J 2012;33:1500–10. 10.1093/eurheartj/ehr488 22246443

[14] Heseltine-Carp W, Courtman M, Browning D, Kasabe A, Allen M, Streeter A et al Machine learning to predict stroke risk from routine hospital data: a systematic review. Int J Med Inform 2025;196:105811. 10.1016/j.ijmedinf.2025.105811 39908727

[15] Lu J, Hutchens R, Hung J, Bennamoun M, McQuillan B, Briffa T et al Performance of multilabel machine learning models and risk stratification schemas for predicting stroke and bleeding risk in patients with non-valvular atrial fibrillation. Comput Biol Med 2022;150:106126. 10.1016/j.compbiomed.2022.106126 36206696

[16] Lip GYH, Genaidy A, Tran G, Marroquin P, Estes C, Sloop S. Improving stroke risk prediction in the general population: a comparative assessment of common clinical rules, a new multimorbid Index, and machine-learning-based algorithms. Thromb Haemost 2022;122:142–50. 10.1055/a-1467-2993 33765685

[17] Huisman MV, Rothman KJ, Paquette M, Teutsch C, Diener HC, Dubner SJ et al Antithrombotic treatment patterns in patients with newly diagnosed nonvalvular atrial fibrillation: the GLORIA-AF registry, phase II. Am J Med 2015;128:1306–13.e1. 10.1016/j.amjmed.2015.07.013 26239094

[18] Huisman MV, Lip GY, Diener HC, Dubner SJ, Halperin JL, Ma CS et al Design and rationale of global registry on long-term oral antithrombotic treatment in patients with atrial fibrillation: a global registry program on long-term oral antithrombotic treatment in patients with atrial fibrillation. Am Heart J 2014;167:329–34. 10.1016/j.ahj.2013.12.006 24576516

[19] Ding WY, Lane DA, Gupta D, Huisman MV, Lip GYH, Investigators G-A. Incidence and risk factors for residual adverse events despite anticoagulation in atrial fibrillation: results from phase II/III of the GLORIA-AF registry. J Am Heart Assoc 2022;11:e026410. 10.1161/JAHA.122.026410 PMC9375480 35876418

[20] Tibshirani R . Regression shrinkage and selection via the lasso. J R Stat Soc Ser B (Methodol) 1996;58:267–88.

[21] Meinshausen N, Bühlmann P. Stability selection. J R Stat Soc Series B Stat Methodol 2010;72:417–73.

[22] Schoenfeld D . Partial residuals for the proportional hazards regression model. Biometrika 1982;69:239–41.

[23] Sullivan LM, Massaro JM, D'Agostino RB Sr. Presentation of multivariate data for clinical use: the framingham study risk score functions. Stat Med 2004;23:1631–60. 10.1002/sim.1742 15122742

[24] Harrell FE Jr, Califf RM, Pryor DB, Lee KL, Rosati RA. Evaluating the yield of medical tests. JAMA 1982;247:2543–6. 7069920

[25] Stone M . Cross-validatory choice and assessment of statistical predictions. J R Stat Soc Ser B (Methodol) 1974;36:111–33.

[26] Chen T, Guestrin C. XGBoost: a scalable tree boosting system. Proceedings of the 22nd ACM SIGKDD International Conference on Knowledge Discovery and Data Mining, 13-17 August 2016. San Francisco: Association for Computing Machinery, 2016. pp. 785–94. doi:10.1145/2939672.2939785

[27] Breiman L . Random forests. Mach Learn 2001;45:5–32.

[28] Pencina MJ, 'Agostino D Sr, Steyerberg RB, W E. Extensions of net reclassification improvement calculations to measure usefulness of new biomarkers. Stat Med 2011;30:11–21. 10.1002/sim.4085 PMC3341973 21204120

[29] Pencina MJ, D'Agostino RB Sr, D’Agostino RB Jr, Vasan RS. Evaluating the added predictive ability of a new marker: from area under the ROC curve to reclassification and beyond. Stat Med 2008;27:157–72; discussion 207–112. 10.1002/sim.2929 17569110

[30] Van Calster B, Nieboer D, Vergouwe Y, De Cock B, Pencina MJ, Steyerberg EW. A calibration hierarchy for risk models was defined: from utopia to empirical data. J Clin Epidemiol 2016;74:167–76. 10.1016/j.jclinepi.2015.12.005 26772608

[31] Steyerberg EW, Vickers AJ, Cook NR, Gerds T, Gonen M, Obuchowski N et al Assessing the performance of prediction models: a framework for traditional and novel measures. Epidemiology 2010;21:128–38. 10.1097/EDE.0b013e3181c30fb2 PMC3575184 20010215

[32] Vickers AJ, Elkin EB. Decision curve analysis: a novel method for evaluating prediction models. Med Decis Making 2006;26:565–74. 10.1177/0272989X06295361 PMC2577036 17099194

[33] Tse HF, Teo WS, Siu CW, Chao T-F, Park H-W, Shimizu W et al Prognosis and treatment of atrial fibrillation in Asian cities: 1-year review of the Asia-Pacific heart rhythm society atrial fibrillation registry. Europace 2022;24:1889–98. 10.1093/europace/euab327 35025986

[34] Boriani G, Proietti M, Laroche C, Fauchier L, Marin F, Nabauer M et al Contemporary stroke prevention strategies in 11 096 European patients with atrial fibrillation: a report from the EURObservational research programme on atrial fibrillation (EORP-AF) long-term general registry. Europace 2018;20:747–57. 10.1093/europace/eux301 29016832

[35] Chang HC, Chan YH, Kuo L, Chao TF. Patient care pathways and risk assessments in patients with atrial fibrillation: a comparison of Asian versus non-Asian cohorts. Thromb Haemost 2025;125:1173–80 10.1055/a-2595-5575 40393656

[36] van der Endt VHW, Milders J, Penning de Vries BBL, Trines SA, Groenwold RHH, Dekkers OM et al Comprehensive comparison of stroke risk score performance: a systematic review and meta-analysis among 6 267 728 patients with atrial fibrillation. Europace 2022;24:1739–53. 10.1093/europace/euac096 PMC9681133 35894866

[37] Fox KAA, Virdone S, Pieper KS, Bassand J-P, Camm AJ, Fitzmaurice DA et al GARFIELD-AF risk score for mortality, stroke, and bleeding within 2 years in patients with atrial fibrillation. Eur Heart J Qual Care Clin Outcomes 2022;8:214–27. 10.1093/ehjqcco/qcab028 PMC8888127 33892489

[38] Hijazi Z, Lindback J, Alexander JH, Hanna M, Held C, Hylek EM et al The ABC (age, biomarkers, clinical history) stroke risk score: a biomarker-based risk score for predicting stroke in atrial fibrillation. Eur Heart J 2016;37:1582–90. 10.1093/eurheartj/ehw054 PMC4875560 26920728

[39] Singer DE, Chang Y, Borowsky LH, Fang MC, Pomernacki NK, Udaltsova N et al A new risk scheme to predict ischemic stroke and other thromboembolism in atrial fibrillation: the ATRIA study stroke risk score. J Am Heart Assoc 2013;2:e000250. 10.1161/JAHA.113.000250 PMC3698792 23782923

