# Supplementary material for: Development and validation of the GLORIA-AF Stroke Weighted Risk Score for patients with atrial fibrillation
Source: Europace. 2026 Aug 29;28(10):euag236. doi: 10.1093/europace/euag236 (PMC13646627; doi:10.1093/europace/euag236)
Supplement: Supplementary file 1 [file euag236_supplementary_data.docx]

**Supplementary Materials**

**Supplementary Methods**

**Supplementary Table 1.** Definitions of Binary-transformed Predictors

**Supplementary Table 2.** Missing Data and Complete-Case Cohort Construction in the GLORIA-AF Derivation Cohort

**Supplementary Table 3.** Integrated Discrimination Improvement and Net Reclassification Improvement in the Derivation and External Validation Cohorts

**Supplementary Table 4.** Baseline Characteristics in the EORP-AF/APHRS-AF External Validation Cohort

**Methods**

Mathematical Formulation of LASSO-penalized Cox Regression with Stability Selection

LASSO-penalized Cox proportional hazards regression was used to select predictors from the prespecified binary candidate variables while accounting for differences in follow-up time and censoring. For patient i, T̃i denotes the observed follow-up time from baseline to the earliest occurrence of stroke, death, loss to follow-up or withdrawal, or administrative censoring at 3 year, and δi denotes the event indicator, with δi = 1 for stroke and δi = 0 otherwise. Under the cause-specific framework, death before stroke was treated as a censoring event.

For the vector of candidate predictors xi, the Cox proportional hazards model was specified as follows:

$$h_{i}(t|\mathbf{x}_{i})=h_{0}(t)\exp({\mathbf{x}_{i}}^{T}\boldsymbol{\beta})$$

where hi(t | xi) denotes the cause-specific hazard of stroke for patient i, h0(t) denotes the unspecified baseline hazard function, and β = (β1, …, βp) denotes the vector of regression coefficients for the p candidate predictors. Defining the linear predictor as

$$\eta_{i}={\mathbf{x}_{i}}^{T}\boldsymbol{\beta}$$

the Cox partial log-likelihood was expressed as

$$\mathcal{l}_{\text{PL}}(\boldsymbol{\beta})=\sum_{i=1}^{n} \delta_{i}[\eta_{i}-\log\{\sum_{k\in R(\tilde{T}_{i})} \exp(\eta_{k})\}]$$

where R(T̃i) denotes the risk set immediately before the observed event time for patient i. The LASSO estimator at penalty parameter λ was obtained by minimizing the negative Cox partial log-likelihood with an L1 penalty:

$$\boldsymbol{\beta}^{\text{LASSO}}(\lambda)=\underset{\boldsymbol{\beta}}{\text{arg min}}\{-\mathcal{l}_{\text{PL}}(\boldsymbol{\beta})+\lambda\left\| \boldsymbol{\beta} \right\|_{1}\}$$

$$\left\| \boldsymbol{\beta} \right\|_{1}=\sum_{j=1}^{p} |\beta_{j}|$$

No conventional intercept was estimated because the unspecified baseline hazard function serves this role in the Cox model.

Stability selection was performed by repeatedly refitting the LASSO-penalized Cox model across B resampled datasets using the same candidate predictors and prespecified penalty-selection procedure. For predictor j, the stability selection probability was calculated as

$$\hat{\pi}_{j}=\frac{1}{B}\sum_{b=1}^{B} I({\beta_{j}}^{\left( b \right)}(\lambda_{b})\neq0)$$

where βj(b)(λb) denotes the fitted LASSO coefficient for predictor j in resampling iteration b, λb denotes the penalty parameter selected in that iteration, and I(·) denotes the indicator function. Predictors satisfying the prespecified stability threshold

$$\hat{\pi}_{j}\geq0.80$$

were retained. All retained predictors were subsequently entered simultaneously into an unpenalized multivariable Cox proportional hazards model fitted in the complete GLORIA-AF derivation cohort. The coefficients from this refitted multivariable Cox model, rather than the penalized LASSO coefficients, were used to derive the integer point weights.

Conversion of Multivariable Cox Regression Coefficients Into Integer Points

Because all predictors retained in the final multivariable Cox model had positive regression coefficients, the full-precision coefficients were linearly rescaled to a prespecified range of 1–5 points. Let βmin and βmax denote the smallest and largest full-precision positive coefficients, respectively. The coefficient interval corresponding to one point was defined as

$$c=\frac{\beta_{\text{max}}-\beta_{\text{min}}}{4}$$

For predictor j, the unrounded point value was calculated as

$${P_{j}}^{*}=1+\frac{\beta_{j}-\beta_{\text{min}}}{c}$$

Equivalently,

$${P_{j}}^{*}=1+4(\frac{\beta_{j}-\beta_{\text{min}}}{\beta_{\text{max}}-\beta_{\text{min}}})$$

The final integer weight was obtained by rounding the unrounded value to the nearest integer:

$$P_{j}=\text{floor}({P_{j}}^{*}+0.5)$$

This transformation assigns 1 point to the predictor with the smallest coefficient and 5 points to the predictor with the largest coefficient, with all remaining coefficients positioned proportionally between these reference values. The transformation was performed using the full-precision coefficients before rounding the coefficients for presentation. No manual modification or post hoc adjustment of individual predictor weights was applied.

For patient i, the total GLORIA-AF Stroke Weighted Risk Score was calculated as

$$S_{i}=\sum_{j=1}^{10} P_{j}X_{ij}$$

where Xij = 1 when risk factor j was present and Xij = 0 when it was absent. The resulting total score ranged from 0 to 23 points, with higher scores indicating a higher predicted 3-year stroke risk.

Calculation of the GLORIA-AF Stroke Weighted Risk Score

The GLORIA-AF Stroke Weighted Risk Score was calculated by summing the integer weights assigned to each baseline risk factor present in an individual patient. Each risk factor contributed its assigned weight when present and contributed 0 points when absent. The total score ranged from 0 to 23 points, with higher scores indicating a higher predicted risk of 3-year stroke.

***GLORIA AF Stroke Weighted Risk Score*** *= 3·I(Age >65 years old) + 5·I(Previous thromboembolism) + 2·I(Smoking history) + 2·I(Persistent or Permanent AF) + 1·I(Coronary artery disease) + 2·I(Hypertension) + 1·I(Hyperlipidaemia) + 4·I(Abnormal kidney function) + 2·I(Diabetes) + 1·I(Heart failure)*

where $I(\cdot)$ denotes an indicator function equal to 1 if the condition is present at baseline and 0 otherwise. AF denotes atrial fibrillation. Detailed definitions of all binary predictors are provided in Supplementary Table 1.

**Supplementary Table 1. Definitions of Binary-transformed Predictors**

| **Binary-transformed predictor**^a^ | **Definition** |
| --- | --- |
| Age group | Age >65 years (reference, ≤65 years) |
| Sex | Female sex (reference, male sex) |
| Heart failure | Presence of congestive heart failure at baseline (reference, absence) |
| Hypertension | History of hypertension or systolic blood pressure >160 mm Hg at baseline (reference, absence) |
| Previous thromboembolism | Previous stroke, transient ischemic attack, or systemic embolism at baseline (reference, absence) |
| Vascular disease | Prior myocardial infarction, peripheral artery disease, or complex aortic plaque at baseline (reference, absence) |
| Diabetes | Diabetes mellitus at baseline (reference, absence) |
| BMI group | Normal body weight, BMI 18.5 to <25 (reference, overweight or obesity with BMI ≥25); patients with BMI <18.5 were excluded |
| Ethnicity | Non-White ethnicity (reference, White ethnicity) |
| Smoking history | Former or current smoking (reference, never smoking) |
| Alcohol intake | Alcohol intake ≥8 drinks/week (reference, <8 drinks/week or no alcohol intake) |
| Type of AF | Persistent or permanent AF (reference, paroxysmal AF) |
| Hyperlipidaemia | Hyperlipidaemia at baseline (reference, absence) |
| Coronary artery disease | Coronary artery disease at baseline (reference, absence) |
| Previous bleeding | History of previous bleeding at baseline (reference, absence) |
| Respiratory disease | History of chronic obstructive pulmonary disease (COPD) at baseline (reference, absence) |
| Abnormal kidney function | Presence of any of the following: 1. Chronic dialysis; 2. renal transplantation; 3. serum creatinine ≥200 *μ*mol/L at baseline (reference, absence) |
| Baseline OAC treatment | No baseline OAC treatment, defined as no NOAC or VKA treatment at baseline (reference, OAC treatment defined as NOAC or VKA treatment at baseline) |

Abbreviations: BMI, body mass index; AF, atrial fibrillation; OAC, oral anticoagulant; VKA, vitamin K antagonist

^a^All candidate predictors were defined at baseline and transformed into binary indicators for model development. Parentheses denote reference categories. Baseline OAC treatment was for baseline characteristics only, and was not included in predictor selection process.

**Supplementary Table 2. Missing Data and Complete-Case Cohort Construction in the GLORIA-AF Derivation Cohort**

| **Variable** | **Missingness, N** | **Missingness, %** |
| --- | --- | --- |
| Stroke outcome/follow-up | 10,431 | 28.49% |
| Age | 642 | 1.75% |
| Sex | 0 | 0.00% |
| heart failure | 329 | 0.90% |
| Hypertension | 0 | 0.00% |
| Previous thromboembolism | 0 | 0.00% |
| Vascular disease | 0 | 0.00% |
| Diabetes | 1 | 0.01% |
| BMI | 443 | 1.21% |
| Ethnicity | 2,196 | 6.00% |
| Smoking history | 1,197 | 3.27% |
| Alcohol consumption | 2,433 | 6.64% |
| Type of AF | 0 | 0.00% |
| Hyperlipidaemia | 961 | 2.62% |
| Coronary artery disease | 939 | 2.56% |
| Previous bleeding | 680 | 1.86% |
| Respiratory disease | 0 | 0.00% |
| Abnormal kidney function | 1 | 0.01% |

Abbreviations: BMI, body mass index; AF, atrial fibrillation; OAC, oral anticoagulant.

The source GLORIA-AF derivation dataset consists of 36,617 patients. After excluding missingness reported in this table, 20,517 patients remained.

**Supplementary Table 3. Integrated Discrimination Improvement and Net Reclassification Improvement in the Derivation and External Validation Cohorts**

| **Measure** | **Estimate (95% CI)** |
| --- | --- |
| **Derivation cohort** |  |
| IDI | 0.0052 (0.0034-0.0071) |
| NRI (event) | 0.1367 (0.0443-0.2239) |
| NRI (non-event) | 0.0463 (0.0262-0.0663) |
| Total NRI | 0.1830 (0.0848-0.2745) |
| Discrimination slope: GLORIA-AF Stroke Weighted Risk Score | 0.0123 |
| Discrimination slope: CHA2DS2-VA | 0.0071 |
| P value of difference in discrimination slopes | <0.001 |
| **External validation cohort** |  |
| IDI | 0.0034 (0.0002-0.0067) |
| NRI (event) | 0.0631 (-0.1401 to 0.2208) |
| NRI (non-event) | 0.1060 (0.0128-0.2743) |
| Total NRI | 0.1691 (-0.0931 to 0.4513) |
| Discrimination slope: GLORIA-AF Stroke Weighted Risk Score | 0.0061 |
| Discrimination slope: CHA2DS2-VA | 0.0027 |
| P value of difference in discrimination slopes | 0.043 |

Abbreviations: IDI, integrated discrimination improvement; NRI, net reclassification improvement; CI, confidence interval.

**Supplementary Table 4. Baseline Characteristics in the EORP-AF/APHRS-AF External Validation Cohort**

| **Characteristic** | **EORP-AF (N=5,670)** | **APHRS-AF (N=2,639)** | **Combined (N=8,309)** |
| --- | --- | --- | --- |
| Age, mean (SD), years | 69.3 (11.2) | 67.6 (11.7) | 68.8 (11.4) |
| Female sex | 2,223 (39.2%) | 912 (34.6%) | 3,135 (37.7%) |
| Heart failure | 2,244 (39.6%) | 559 (21.2%) | 2,803 (33.7%) |
| Hypertension | 3,662 (64.6%) | 1,648 (62.4%) | 5,310 (63.9%) |
| Previous thromboembolism | 672 (11.9%) | 319 (12.1%) | 991 (11.9%) |
| Vascular disease | 1,244 (21.9%) | 202 (7.7%) | 1,446 (17.4%) |
| Diabetes | 1,334 (23.5%) | 614 (23.3%) | 1,948 (23.4%) |
| BMI, mean (SD), kg/m2 | 28.2 (5.0) | 25.1 (4.2) | 27.2 (5.0) |
| Non-White ethnicity | 315 (5.6%) | 2,626 (99.5%) | 2,941 (35.4%) |
| Former or current smoking | 1,978 (34.9%) | 696 (26.4%) | 2,674 (32.2%) |
| Alcohol abuse | 391 (6.9%) | 129 (4.9%) | 520 (6.3%) |
| Persistent or permanent AF | 3,849 (67.9%) | 1,447 (54.8%) | 5,296 (63.7%) |
| Hyperlipidaemia | 2,487 (43.9%) | 1,036 (39.3%) | 3,523 (42.4%) |
| Coronary artery disease | 1,619 (28.6%) | 534 (20.2%) | 2,153 (25.9%) |
| Previous bleeding | 354 (6.2%) | 202 (7.7%) | 556 (6.7%) |
| Respiratory disease | 508 (9.0%) | 68 (2.6%) | 576 (6.9%) |
| Baseline OAC treatment | 4,992 (88.0%) | 2,230 (84.5%) | 7,222 (86.9%) |

Abbreviations: SD, standard deviation; BMI, body mass index; AF, atrial fibrillation; OAC, oral anticoagulant.
